# Mechanotransduction and Adrenergic Stimulation in Arrhythmogenic Cardiomyopathy: An Overview of *in vitro* and *in vivo* Models

**DOI:** 10.3389/fphys.2020.568535

**Published:** 2020-11-12

**Authors:** Giorgia Beffagna, Elena Sommariva, Milena Bellin

**Affiliations:** ^1^Department of Cardio-Thoraco-Vascular Sciences and Public Health, University of Padua, Padua, Italy; ^2^Department of Biology, University of Padua, Padua, Italy; ^3^Vascular Biology and Regenerative Medicine Unit, Centro Cardiologico Monzino IRCCS, Milan, Italy; ^4^Veneto Institute of Molecular Medicine, Padua, Italy; ^5^Department of Anatomy and Embryology, Leiden University Medical Center, Leiden, Netherlands

**Keywords:** arrhythmogenic cardiomyopathy, mechanotransduction, adrenergic signaling, cell models of disease, animal models

## Abstract

Arrhythmogenic Cardiomyopathy (AC) is a rare inherited heart disease, manifesting with progressive myocardium degeneration and dysfunction, and life-threatening arrhythmic events that lead to sudden cardiac death. Despite genetic determinants, most of AC patients admitted to hospital are athletes or very physically active people, implying the existence of other disease-causing factors. It is recognized that AC phenotypes are enhanced and triggered by strenuous physical activity, while excessive mechanical stretch and load, and repetitive adrenergic stimulation are mechanisms influencing disease penetrance. Different approaches have been undertaken to recapitulate and study both mechanotransduction and adrenergic signaling in AC, including the use of *in vitro* cellular and tissue models, and the development of *in vivo* models (particularly rodents but more recently also zebrafish). However, it remains challenging to reproduce mechanical load stimuli and physical activity in laboratory experimental settings. Thus, more work to drive the innovation of advanced AC models is needed to recapitulate these subtle physiological influences. Here, we review the state-of-the-art in this field both in clinical and laboratory-based modeling scenarios. Specific attention will be focused on highlighting gaps in the knowledge and how they may be resolved by utilizing novel research methodology.

## Introduction

Arrhythmogenic cardiomyopathy (AC) is a rare disease, which commonly manifests during late childhood or adolescence with malignant arrhythmias and causes sudden cardiac death (SCD) in otherwise healthy young individuals (Thiene et al., 1988; Thiene and Basso, 2001; Basso et al., 2009). Progressive fibro-fatty replacement of the myocardium is the histopathological hallmark of the disease, although in the early concealed stages, electrophysiological changes may precede structural changes (Basso et al., 1996; Kaplan et al., 2004b; Bauce et al., 2005; Gomes et al., 2012; Rizzo et al., 2012). A major breakthrough in the understanding of AC came with the realization that this disease is associated with mutations in desmosomal proteins (Gerull et al., 2004; Yang et al., 2006), resulting in impaired mechanical properties of cardiac cells. This discovery fueled several studies aimed at uncovering the relationship between desmosome abnormalities and AC pathological and clinical findings (Thiene, 2015).

The heart is indeed constantly challenged by mechanical stress and both its function and mechanical integrity strictly depends on correct cell-cell and cell-extracellular matrix (ECM) connections. Desmosomes are junctional complexes that interconnect adjacent cells, and therefore are highly expressed in tissues subject to mechanical stretch and load, such as skin and heart. Together with integrins and cadherins, desmosomes are coupled to structural mechanoresponsive cytoskeletal elements, such as F-actin, microtubules, and intermediate filaments, which allow cardiomyocytes (CMs) to adapt to external and internal physical stimuli. These mechanoresponsive elements are necessary for correct dissipation of mechanical loads and for efficient mechanotransduction. Mechanotransduction is the mechanism by which force transmission between cells and between cell-ECM is translated into a series of dynamic intracellular signaling events (Hoffman et al., 2011). In addition, cell shape can influence cell lineage commitment through ROCK-mediated cytoskeletal tension (McBeath et al., 2004), since cell-cell and cell-ECM adhesions translate into soluble intracellular signals, influencing cell fate.

In AC, genetic defects in desmosomes and other mechanosensitive or mechanotransduction proteins lead to altered response to physiologic mechanical load and even more to exercise. In AC, mechanical load causes intracellular signaling changes (mainly in Wnt/βcatenin and Hippo pathways) driving alternative cell fate, such as fibrotic or adipogenic signaling.

In addition, competitive sports expose the heart to adrenergic stress, which may lead to electrical and functional destabilization. Indeed, exercise is one of the main triggers for life-threatening arrhythmias and SCD in several inherited heart conditions, including AC (Furlanello et al., 1998; Firoozi et al., 2002; Thiene et al., 2016; Corrado and Zorzi, 2018), as recently demonstrated by the tragic events of top-level athletes (Catto et al., 2019).

Once the genetic causes of AC were discovered, several *in vitro* and *in vivo* models were quickly established. Cell models include CMs, the primary cell type affected by AC-linked mutations, and non-CM cell types, and both human and non-human models. *In vivo* models mainly include transgenic mice and, to a lesser extent, zebrafish knock-down models and the spontaneous AC feline and canine models were studied. Both *in vitro* and *in vivo* models helped to reveal the major pathogenic mechanisms underlying AC. However, in AC, the genetic substrate is not sufficient for a comprehensive disease modeling. Therefore, different mechanical and adrenergic stimuli have been applied to cell and animal models to mimic the effect of exercise on AC pathogenesis and to understand their finer molecular determinants. This aspect is the main focus of this review.

## Clinical Aspects of Mechanical Load and Adrenergic Stress in AC Patients

### Most AC Causal Genes Have Mechanic and Adrenergic-Response Functions

Cardiac tissue is constantly exposed to different external forces such as mechanical load and stretch, in addition to intrinsic forces from the contraction machinery of single CMs. These extrinsic and intrinsic forces contribute to tissue morphogenesis, homeostasis, and regeneration and affect different aspects, such as cell size and shape, proliferation, differentiation, and migration (Evans et al., 2013).

Cardiac output and rhythm are tightly regulated by the autonomic nervous system (Silvani et al., 2016). Adrenergic nerves are in contact with cardiac cells (Kawashima, 2005) and signal transmission is based on neuro-cardiac synapses. The stimulation signals include the release of the adrenergic hormones catecholamines (noradrenaline and adrenaline), which in turn are sensed by cardiac cells through adrenoceptors, resulting in a positive inotropic response of the heart (Mary-Rabine et al., 1978; Hedberg et al., 1985; Chamberlain et al., 1999).

**FIGURE 1:**
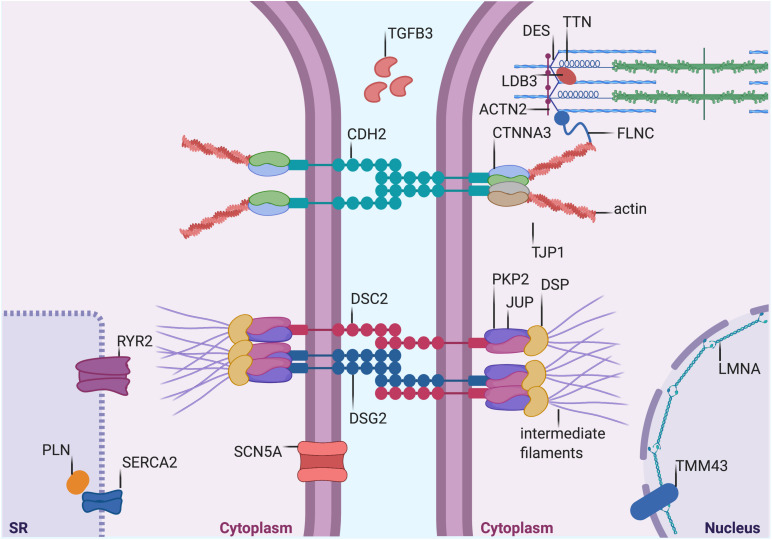
Proteins mutated in AC. Graphical representation of proteins that are mutated in AC. Both proteins desmosomal (see Table 1) and non-desmosomal proteins (see Table 2) are depicted in this figure. DSP, desmoplakin; PKP2, plakophilin-2; DSG2, desmoglein-2; DSC2, desmocollin-2; JUP, plakoglobin; CTNNA3, α-T-Catenin; CDH2, cadherin-2; TJP1, tight Junction Protein ZO-1; DES, desmin; TTN, titin; FLMN, filamin C; LMNA, lamin A/C; TMEM43, transmembrane protein-43; ACTN2, α-actinin-2; LDB3, lim domain binding 3 or ZASP; RYR2, ryanodine Receptor-2; PLN, phospholamban; TGFβ3, transforming growth factor β3; and SCN5A, sodium voltage-gated channel alpha subunit 5. SERCA2, sarcoplasmic/endoplasmic reticulum calcium ATPase 2. The figure was redrawn from Austin et al., 2019.

Arrhythmogenic cardiomyopathy is an inherited cardiomyopathy characterized by a high degree of genetic heterogeneity (Celeghin and Pilichou, 2019). To date, many genes have been associated with the disease, although some very rarely. Tables 1, 2 summarize the desmosomal and non-desmosomal genes, respectively, for which a causative role in AC has reached consensus and the corresponding proteins are graphically represented in Figure 1. However, additional genes were associated to AC and are reviewed elsewhere (Towbin et al., 2019). Interestingly, some of these genes are associated to more than one channelopathy or cardiomyopathy (e.g., *LMNA*, *SCN5A*, and *TITIN*), and other genes (e.g., *RYR2*) display phenotypical overlap with other cardiac diseases which raises questions regarding their association to AC. The more likely scenario is that the concept of one gene-one disease paradigm does not hold (Cerrone et al., 2019). Importantly, although mutations in the *RYR2* gene were initially recognized in phenocopy AC (Tiso et al., 2001), they rather belong to the morbid entity clinically reported by Philippe Coumel in Paris, characterized by effort-induced polymorphic ventricular arrhythmias and SCD with a structurally normal heart, later named catecholaminergic polymorphic ventricular tachycardia (Prof. G. Thiene, personal communication). Nevertheless, the majority of genes involved in AC encode for proteins related to cardiac tissue extrinsic and/or intrinsic forces (Tables 1, 2), making stretch and mechanosensing of fundamental importance for understanding the pathogenic mechanisms of AC (Padrón-Barthe et al., 2019). In addition, some AC-associated genes have adrenergic-dependent functions (Tables 2, making AC phenotypes vulnerable to autonomic signals (Shen and Zipes, 2014).

**TABLE 1:** Desmosomal genes associated to AC.

Gene	Chromosomal location	Protein	Prevalence	Mainly associated to:	References
*DSP*	6p24	Desmoplakin	10–15%	AC	Rampazzo et al., 2002
*PKP2*	12p11	Plakophilin-2	10–45%	AC	Gerull et al., 2004
*DSG2*	18q12	Desmoglein-2	7–10%	AC	Pilichou et al., 2006
*DSC2*	18q12	Desmocollin-2	Rare	AC	Syrris et al., 2006; Beffagna et al., 2007
*JUP*	17q23	Plakoglobin	Rare	AC	McKoy et al., 2000

**TABLE 2:**
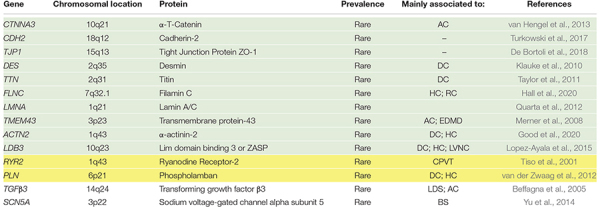
Non-desmosomal genes also associated with AC phenocopies.

*Green, genes with mechanical function; yellow, genes with a function in response to adrenergic stimuli. Association based on Online Mendelian Inheritance in Man catalog. AA, Aortic Aneurism; AC, Arrhythmogenic Cardiomyopathy; BS, Brugada Syndrome; CPVT, Catecholaminergic Polymorphic Ventricular Tachycardia; LDS, Loeys-Dietz Syndrome; DC, Dilated Cardiomyopathy; HC, Hypertrophic Cardiomyopathy; RC, Restrictive Cardiomyopathy; ED-MD, Emery-Dreifuss Muscular Dystrophy; and LVNC, Left Ventricular Non-Compaction.*

About 50% of AC probands are carriers of mutations in genes encoding desmosomes (Pilichou et al., 2016). Desmosomes are intercellular junctions composed of three protein families essential for mediating strong intercellular cohesion (Garrod, 2010; Green et al., 2010; Kowalczyk and Green, 2013). These three protein families are: (i) desmosomal cadherins (ii) armadillo proteins and (iii) plakins. The desmosomal cadherins, named desmogleins (DSGs) and desmocollins (DSCs), are transmembrane proteins whose extracellular domains form the adhesive interface of the desmosome, whereas their cytoplasmic tails anchor the armadillo proteins to the desmosomal plaque. The armadillo proteins, plakoglobin (JUP) and plakophilins 1–3 (PKP1-3) in turn, bind to desmoplakin (DSP), a member of the plakin family of cytoskeleton-associated proteins. DSP links the desmosome to the desmin filament network which is essential to provide tensile strength (Hatzfeld et al., 2017). All genes encoding desmosomal proteins are involved in the genetic determination of AC (Celeghin and Pilichou, 2019).

Autosomal dominant is the most common pattern of inheritance in AC but desmosomal recessive forms have also been reported to present a cardio-cutaneous phenotype, named Naxos disease (Kaplan et al., 2004b) when *JUP* carries a homozygous mutation (McKoy et al., 2000), and Carvajal syndrome when *DSP* carries a homozygous mutation (Carvajal-Huerta, 1998; Norgett et al., 2000). Further analysis of the structural and molecular pathology of the heart in Carvajal syndrome showed a distinct cardiomyopathy characterized by focal ventricular aneurysms and reduced expression of desmosomal proteins DSP and plakoglobin, and the gap junction protein connexin 43 (CX43) at intercalated disks. These abnormal protein–protein interactions cause both contractile and electrical dysfunction in Carvajal syndrome (Kaplan et al., 2004a).

Two adherens junction proteins, N-cadherin (CDH2; Mayosi et al., 2017) and αT-catenin (CTNNA3; van Hengel et al., 2013), have been associated with AC, that broadens cell-cell adhesion disease pathogenesis beyond the desmosomes. In the extracellular space, cadherins of adjacent cells bind together, while the cytoplasmic domains of cadherins are linked to the actin cytoskeleton through β-catenin, plakoglobin, and αT-catenin (Janssens et al., 2001; Kobielak and Fuchs, 2004). Since N-cadherin and αT-catenin co-localize with the area composita and not desmosomes, AC could also be considered as an “area composita disease” rather than a classical “desmosomal disease” (van Hengel et al., 2013). In addition, variants in the tight junction protein zonula occludens-1 (*TJP1*) gene have recently been described in AC cases (De Bortoli et al., 2018). The translation product of *TJP1*, ZO-1 is an adapter protein that interacts with gap junctions and area composita proteins and plays a crucial role in the cardiac functional syncytium (Zhang et al., 2020).

Junctional complexes mediate cell-cell adhesion tethering adjacent cells and anchoring them to the ECM (Gumbiner, 1996). Junctional proteins provide tensile strength and resilience to the tissue as well as mechanical, electrical, and chemical continuity between cells. This function is of particular importance for cardiac cells, allowing the mechanical work of individual myocytes to integrate into the pumping function of the heart, continuously subjected to contraction and relaxation cycles of different intensity (McCain et al., 2012). Moreover, desmosomes and adherens junctions constitute a mechanotransduction hub (Pannekoek et al., 2019). AC-causing mutations that modify junctions might thus alter cardiac cell tethering or signaling functions (Hariharan et al., 2014).

The cytoplasmic face of desmosomes and adherens junctions are connected to cytoskeleton proteins, e.g., actin. Mutations in some cytoskeleton-associated proteins (Titin or TTN, Filamin or FLNC, and Desmin or DES) were also found in some AC patients (Klauke et al., 2010; Taylor et al., 2011; Hall et al., 2020). Moreover, variants in two proteins of the sarcomere stabilizing the Z-line, namely α-actinin-1 (ACTN1; Good et al., 2020), which is a cytoskeleton protein binding actin filaments and stabilizing the contractile apparatus, and lim domain binding 3 (LDB3 or ZASP; Lopez-Ayala et al., 2015), which is an α-actinin interacting protein, have also been described in AC patients. Cytoskeleton proteins are in charge of structural preservation and mechanotransduction.

Similarly, transmembrane protein 43 (TMEM43) and Lamin A/C (LMNA), both involved in preserving the structural integrity continuum at the nuclear membrane level, are rarely mutated in AC (Merner et al., 2008; Quarta et al., 2012).

Calcium (Ca^2+^) handling proteins, such as PLN and RYR2, regulating Ca^2+^ storage in the sarcoplasmic-endoplasmic reticulum of cardiac cells, have been implicated in AC pathogenesis (Tiso et al., 2001; van der Zwaag et al., 2012). They are involved in regulating excitation-contraction coupling and many Ca^2+^-dependent functions. Ca^2+^ is the major second messenger mediating response to sympathetic stimuli through beta adrenergic receptor activation, cyclic AMP (cAMP) formation and Ca^2+^-dependent kinases activation (de Lucia et al., 2018). Furthermore, through the action of stretch-activated channels, mechanical forces are transduced into ion fluxes including Ca^2+^ (Martinac, 2004).

Interestingly, positron emission tomography showed that the AC myocardium displayed reduced β-adrenergic receptor density, which was hypothesized to be the result of a secondary downregulation in response to either local increased firing rates or impaired presynaptic catecholamine reuptake in efferent sympathetic nerves (Wichter et al., 2000). These data suggest a link between sympathetic hyperactivity and life-threatening arrhythmias triggering in patients with AC.

Despite such deep understanding of AC-linked genetics, AC diagnosis is mainly based on clinical parameters and it remains challenging to be unambiguously identified due to non-specific clinical features and the variable clinical presentation of the disease. Diagnostic criteria were established by an international task force in 1994 and revised in 2010 due to the use of emerging diagnostic modalities and the discovery of genes involved in AC (McKenna et al., 1994; Marcus et al., 2010). Recently, the criteria have been better defined and widened to include left dominant AC and pediatric cases (Corrado et al., 2020). However, once a causative mutation is identified in a proband, predictive genetic testing is advisable for relatives, in order to adopt either clinical follow-up or preventive strategies, such as sport restriction and beta-blockers or ICD therapy in individuals at risk (Corrado et al., 2015; Stadiotti et al., 2019).

### AC Penetrance Is Dependent on Physical Exercise

Exercise is associated with modulation of cardiac sympathetic activation. As a consequence, aerobic exercise promotes beneficial effects on the treatment of diseases such as arterial hypertension, atherosclerosis, venous insufficiency, and peripheral arterial disease (Leosco et al., 2013). However, a small but significant proportion of athletes die suddenly (Corrado and Zorzi, 2018), with exercise being particularly deleterious in AC. Despite its genetic determinants, AC penetrance is largely affected by high-intensity physical activity. While the most accepted view is that exercise enhances a genetic predisposition, others theorize that genetic and environmental stressors may combine to variable extent resulting in overt disease with inconsistent phenotype severity (Prior and La Gerche, 2020).

Undoubtedly, exercise is a strong predictor of life-threatening ventricular arrhythmias in AC (Lie et al., 2018b). Accordingly, the risk of SCD was increased five-fold in AC athletes (Corrado et al., 2003) and arrhythmic events often occurred during exercise (Thiene et al., 1988).

For this reason, some countries worldwide, supported by expert consensus of the major cardiology societies, have defined a pre-participation screening program for competitive athletes, in order to avoid the combination of intense exercise and the presence of underlying cardiomyopathy (among which AC; Maron et al., 2015; Mont et al., 2017). This has drastically reduced the occurrence of SCD (Corrado et al., 2006), as ECG alone can effectively recognize most individuals requiring sport disqualification (Mont et al., 2017). However, management and costs of the screening do not allow a thorough analysis on such a large population and the transition to secondary tests, including genetics, does improve the recognition yield of individuals at risk (Limongelli et al., 2020).

In clinical settings, exercise testing can be used to mimic, under controlled circumstances, the effect of exercise. Such tests could reveal latent electrocardiogram abnormalities and electrical instability in a significant number of asymptomatic AC gene carriers (Perrin et al., 2013).

**FIGURE 2:**
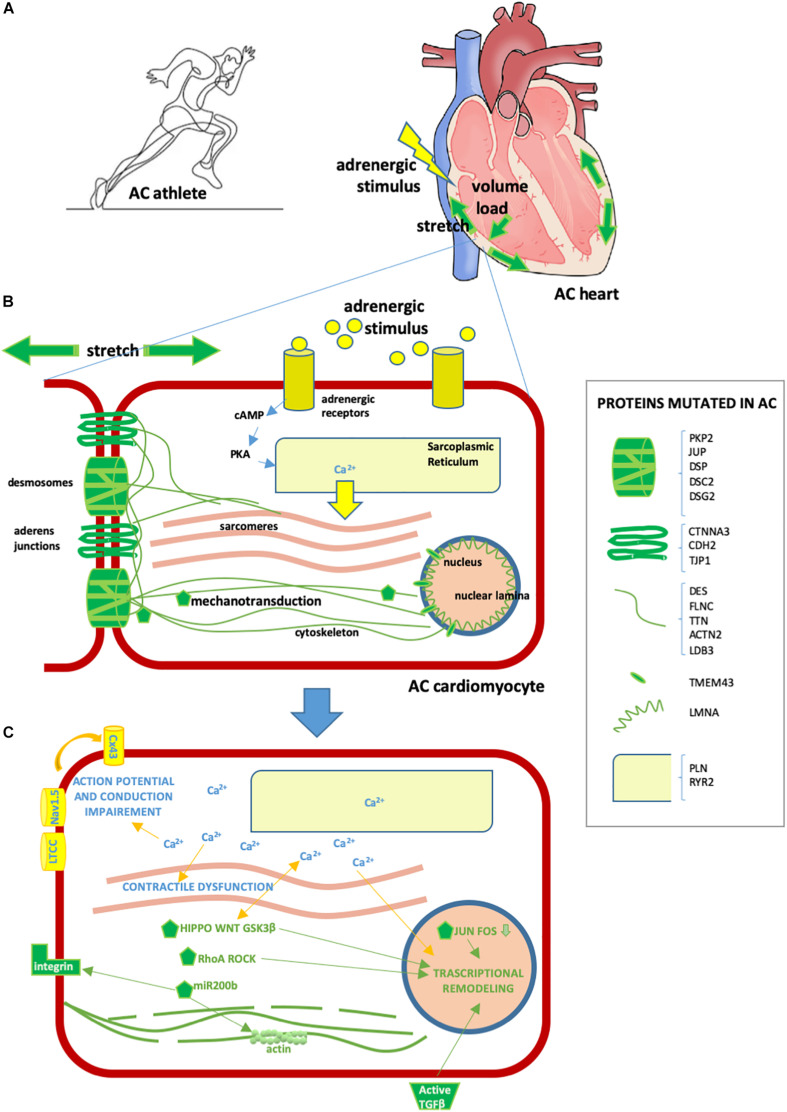
Effect of exercise on the AC heart. **(A)** Exercise involves hyperactivation of adrenergic stimuli and augmented mechanical forces, including stretch and volume load. These physiological mechanisms can exacerbate AC heart remodeling and arrhythmia predisposition, already induced by AC mutations. **(B)** AC cardiomyocyte. In yellow/light blue, adrenergic stimuli or proteins implicated in adrenergic response [some proteins regulating calcium (Ca^2+^) fluxes can be mutated in AC, as detailed in the box on the right]; in green, stretch or mechanical stretch responsive proteins (some proteins implicated in mechanical force management can be mutated in AC, as detailed in the box on the right). **(C)** Consequences of cytoplasmic Ca^2+^ excess and activation of mechanotransduction molecules in AC cardiomyocytes are as follows: – contractile dysfunction due to excitation-contraction coupling defect; – transcriptional remodeling including cell fate change, apoptosis, pro-inflammatory, pro-adipogenic and pro-fibrotic program activation; – intercalated disk remodeling with abnormal activation of the NaV1.5 channel and other Ca^2+^-dependent ion channels, leading to action potential shortening; – lateralization of CX43 and altered permeability leading to conduction defects; – cytoskeleton alteration/disruption, integrins and TGFβ activation. Some of these consequences are mediated by several Ca^2+^-sensitive or mechano-sensitive pathways (namely Hippo, WNT, GSK3β, RhoA ROCK, JUN-FOS, miR-200b, TGFβ, ion channels, and CX43 modulation) which combine with those already genetically active in AC, resulting in a strong additive effect.

Strenuous exercise increases both mechanical load (stretch and volume) and adrenergic stress in humans, the two types of stimulation that AC patients are particularly vulnerable to (Figure 2).

The arrhythmogenic effect of exercise on AC hearts is likely due to sympathetic induction, which, *per se*, can affect electrophysiological mechanisms of arrhythmia initiation and/or maintenance (Johnson and Antoons, 2018). β-adrenergic receptor activation increases sarcoplasmic reticulum Ca^2+^ load, triggering pro-arrhythmogenic effects, and in the AC setting can exacerbate existing Ca^2+^ defects (Moccia et al., 2019). This is supported by the fact that isoproterenol testing can sensibly unveil arrhythmias in AC patients, particularly in the early concealed stages of the disease (Denis et al., 2014).

Additionally, different studies have associated intense endurance exercise with earlier onset and more severe expression of AC disease substrate phenotype (La Gerche, 2015), broadening the effect of sport beyond arrhythmic burden. In AC mutation carriers with a history of endurance exercise, symptoms occur at a younger age than in sedentary individuals, and athletes are more likely to develop heart failure and to meet Task Force criteria for AC (James et al., 2013). Moreover, imaging studies revealed that left and right ventricular function was reduced and right ventricle volume enlarged in athletes when compared to non-athletes (Sen-Chowdhry et al., 2007). Interestingly, an apparent direct dose-response relationship has been established between exercise intensity and severity of AC phenotypes in AC-mutation carriers, where exercise intensity is more damaging than exercise duration (Lie et al., 2018a).

Intense exercise increases ventricular wall stress and the forces to which desmosomes, adherens junctions and the whole cytoskeletal continuum are subjected. In the AC context, defects in these proteins provide susceptibility to mechanical damages and/or alter signal mechanotransduction. Interestingly, AC phenotype may involve both the right and left ventricles with variable expressivity, but the associated mechanisms that are still not well understood. Intriguingly, physiological difference between right and left ventricle thickness and wall tension might result in different mechanotransduction signaling (Mestroni and Sbaizero, 2018); being the right ventricle wall more volume-loaded (La Gerche et al., 2011), this provides an explanation for the predominant right ventricle involvement in both arrhythmias and fibro-fatty remodeling in AC (Thiene and Marcus, 2013). Indeed, the observation that the thin walled right ventricle and the thinnest segment of the left ventricle (posterior wall) are most often involved may reflect these areas being more vulnerable to physical stress or stretch, where, according to Laplace’s law, wall tension is particularly high (Thiene and Marcus, 2013). Accordingly, right ventricular strain measured by echocardiography is associated with worst structural degeneration in AC patients (Malik et al., 2020).

### AC Therapies Include Beta Blocker Administration and Exercise Limitation

Therapeutic options for AC are aimed both at prevention of SCD and at limiting heart failure and symptoms. They mainly consist on lifestyle changes, pharmacological treatment, catheter ablation, implantable cardioverter defibrillator graft, and, in the worst cases, heart transplantation.

Lifestyle changes consist of exercise restrictions, which effectively limited disease progression and reduced the likelihood of sustained ventricular arrhythmias (Calkins et al., 2017). The main pharmacological therapy used with AC patients is β-blocker administration. According to the 2017 AHA/ACC/HRS guidelines for the management of ventricular arrhythmias, a strong recommendation is made both for the use of β-blockers and for avoiding intense exercise (Al-Khatib et al., 2018). While these recommendations and approaches are based on several well-structured studies deemed effective in clinical practice, no systematic randomized studies are available to date.

β-blockers are competitive antagonists of endogenous catecholamines that block receptor sites on adrenergic beta receptors. By binding stress hormone receptors, they weaken the fight-or-flight reaction (Szentmiklosi et al., 2015). Bursts of adrenergic stimulation promote hyperphosphorylation of RYR2 in the myocardium, leading to excess Ca^2+^ influx into the cytoplasm which contributes to electrical instability, thus triggering arrhythmias (Landstrom et al., 2017). This mechanism is particularly relevant for catecholaminergic ventricular tachycardia (CPVT), where arrhythmia occurs in the context of a structurally-normal heart and only in response to adrenergic stress, thus supporting the essential role of β-blockade therapy in these patients. In addition, excessive catecholamine activity is responsible for a number of deleterious effects on the heart, including increased oxygen demand, propagation of inflammatory mediators, and abnormal cardiac tissue remodeling, all of which decrease the efficiency of cardiac contraction and contribute to heart failure progression (de Lucia et al., 2018). The antiarrhythmic of effects β-blockers are based on counteracting the effects of adrenergic stress by: (i) slowing down the heart rate, decreasing spontaneous depolarization of pacemaker cells of the sinus node; (ii) slowing atrio-ventricular conduction, which increases the refractory period of the atrio-ventricular node; (iii) reducing myocardial contractility, thus preventing action potential (AP) duration shortening of myocardial cells; (iv) suppressing catecholamine-induced hypokalemia (Gorre and Vandekerckhove, 2010). Importantly, ventricular structure and function can be modulated by β-blockade. Myocardial ischemia can be reduced though vasodilation, and heart failure can be reduced by modulating gene expression, ultimately inhibiting myocardial oxidative stress and apoptosis (López-Sendón et al., 2004).

Arrhythmogenic Cardiomyopathy guidelines and expert consensus documents generally advise β-blocker use for AC, without indication about β-receptor selectivity (Al-Khatib et al., 2018; Towbin et al., 2019). Indeed, no randomized studies have been performed on specific β-blockers in AC. Recent advances in the pharmacological selectivity of β-blockers has defined three generations: first-generation β-blockers, non-selective, blocking both β1 and β2 receptors, thus acting also in non-cardiac sites; second-generation β-blockers, more cardio-selective since they present higher affinity for β1-receptors; third-generation β-blockers with selectivity for β1 receptors and α1-adrenoreceptors while activating β3-adrenergic receptors. The latter group showed vasodilation, antioxidant, antihypertrophic, and antiapoptotic activities (do Vale et al., 2019). In principle, third-generation β-blockers may be more appropriate for AC by potentially targeting other AC dysfunctions. Accordingly, the MADIT-CRT trial showed that the third generation β-blocker carvedilol was the most effective agent that reduced the number of inappropriate ICD shocks for patients who received an ICD (Ruwald et al., 2013). Also, a case report suggests that carvedilol improved left ventricular function in an AC patient (Hiroi et al., 2004). Specific clinical studies in AC patients are needed to unveil the most effective class of β-blocker.

## *In vitro* AC Cellular Models

Arrhythmogenic Cardiomyopathy cellular models represent extremely useful tools to recapitulate AC-specific traits *in vitro*, to investigate cellular and molecular mechanisms involved in disease onset and progression, and in testing potential treatments. In this section, we focus on the cellular models that supported the link between AC and either mechanical load or adrenergic signaling (Table 3). A complete overview of the cellular models used to study AC have been previously reviewed (Sommariva et al., 2017).

**TABLE 3:** Animal and cellular AC models linking mechanical load/mechanotransduction and adrenergic stimulation with AC pathogenesis.

Model		Gene/Mutation	Phenotype	Type of stress	Conclusions	References
Animal models	Murine	*Dsp* / Cardiac-specific p.R2834H Dsp overexpression	Ventricular enlargement and biventricular cardiomyopathy at rest; right ventricle dilation and focal fat infiltration in response to exercise	Physical exercise (daily running regimen for 12 week)	DSP expression in cardiomyocytes contributes to maintaining cardiac tissue integrity; exercise accelerates cardiac remodeling	Yang et al., 2006; Martherus et al., 2016
		*Dsp* / cardiac-specific Dsp-KO	No phenotype within 2-months after birth. By 6 months of age, cardiac systolic dysfunction and mild myocardial fibrosis. By 1-month increased mortality, cardiac systolic dysfunction and exercise-inducible ventricular arrhythmias.	Early and long treadmill exercise	Treadmill exercise restored transcript levels of dysregulated genes in cardiomyocytes, reducedmyocardial apoptosis, and induced cardiac hypertrophy without affecting cardiac dysfunction	Garcia-Gras et al., 2006; Cheedipudi et al., 2020
		*Dsp* / Cardiomyocyte-specific Dsp-KO	Ultrastructural defects in desmosomal integrity and cardiomyopathy; cell death and fibro-fatty replacement; biventricular dysfunction, failure and death; arrhythmias	Physical exercise (horizontal treadmill at incrementally faster running speeds). Adrenergic stress (intraperitoneal injection of high or low dose epinephrine)	Exercise causes catecholamine-induced arrhythmias	Lyon et al., 2014
		*Pkp2* deficiency	Flecainide and exercise-induced arrhythmia and cardiac remodeling	One-month voluntary running on a treadmill	Exercise-induced pro-arrhythmic behavior due to impaired Ca^2+^ cycling and electrical conduction	Grossmann et al., 2004; van Opbergen et al., 2019
		*PKP2* / card-ac-specific p.R735* overexpression	No AC phenotype at rest; strenuous swimming induced right ventricular dysfunction	Endurance exercise training (10 months strenuous swimming protocol)	Endurance training triggers AC-like phenotype in mice	Cruz et al., 2015
		*Dsg2* / exons 4 and 5 deletion	No phenotype at rest; myocyte injury and redistribution of intercalated disk proteins in response to exercise	Starting from 3 weeks of age, gradually incremented exercise training (swimming)	Exercise reduces survival of Dsg2 mutant mice	Chelko et al., 2016
		*Dsc2* / Embryonic Dsc2-knock-down	Reduction in the desmosomal plaque area, loss of desmosome extracellular electron-dense midlines, and myocardial contractility defects	–	Dsc2 is necessary for normal myocardial structure and function	Heuser et al., 2006
		*Jup* / Jup-KO	Genetic background-dependent embryonic lethality (heart defects) or late-embryonic/perinatal lethality (cardiac dysfunction and severe skin phenotype); thin and detached epidermis; altered physical properties of the skin	Mechanical stress (skin rubbing and cutting)	Jup mutations are responsible of different skin physical properties and susceptibility to mechanical stress	Bierkamp et al., 1996; Bierkamp et al., 1999
		*Jup* / 2057del2 heterozygous mutation	No phenotype at rest; myocyte injury and redistribution of intercalated disk proteins in response to exercise	Starting from 3 weeks of age, gradually exercise training increment (swimming)	Exercise reduces survival of Jup mutant mice	Chelko et al., 2016
		*PLN* / Cardiac-specific heterozygous expression of PLN Arg14Del	By middle age, *het* individuals developed left ventricular dilation, contractile dysfunction, and episodic ventricular arrhythmias, with overt heart failure; cardiomyopathy and heart failure upon adrenergic stimulation	Adrenergic stimulation (chronic suppression of either basal SERCA2a activity or the stimulatory effect of the β-adrenergic signaling pathway)	Chronic suppression of SERCA2 may lead to premature death	Haghighi et al., 2006
	Zebrafish	*Dsp* / transient Dsp-knock-down	Mild developmental delay, signs of microcephaly, pericardial effusion, and decreased heart rate	–	Wnt/β-catenin and Hippo are the final common pathways underlying different desmosomal AC forms	Giuliodori et al., 2018
		*Pkp2* / Pkp2-konck-down	Decreased heart rate, reduced number of intercalated disks, increased intracellular space	–	Pkp2 has structural and signaling roles in heart development	Moriarty et al., 2012
		*Jup* / Jup-knock-down	Reduced number of adhesion junction proteins	–	Loss of Jup leads to altered desmosome structure. Jup antagonizes β-catenin signaling in the heart	Martin et al., 2009
		*JUP* / cardiomyocyte-specific over-expression c.2057del2	Heart enlargement with marked thinning of atrial and ventricular walls, cachexia, and peripheral edema	–	Mortality, reduced I_Na_ current density. SB216763 rescued the AC phenotypes	Asimaki et al., 2014
		*STRN* / 8 bp deletion in the 3’ UTR	Syncope or SCD or heart failure	Exposure to risk factors similar to those of humans may be one of the reasons for spontaneous AC occurrence	STRN co-localizes with desmosomal proteins and interferes with the Wnt pathway thus STRN is a potential contributor to mechanotransduction and downstream sympathetic signaling	Meurs et al., 2010; Oxford et al., 2014; Cattanach et al., 2015
	Canine	Spontaneous model, genetic alterations were unknown	Cardiomyopathy that closely mimics AC in humans	Exposure to risk factors similar to those of humans	Spontaneous canine model is s useful tool to study pathophysiological mechanisms in AC	Fox et al., 2000
	Feline	*PKP2* / c.2386T > C (p.C796R)	Unstable PKP2 proteins that fail to interact with DSP and undergo targeted degradation involving calpain and other proteases	–	PKP2 mutations induce loss of function effects by intrinsic instability and calpain protease- mediated degradation	Kirchner et al., 2012
Cellular models	HL-1	*Pkp2* knock-down	Changes in cytoskeleton organization including perturbation of the actin network and focal adhesions, decreased stiffness, reduced work of detachment	Mechanical load	Pkp2 mutation impact on cardiomyocyte-ECM interactions; miR200b is one of the mediators	Puzzi et al., 2019
		*Pkp2* knock-down	Increased separation of microtubules from the cell extremities	–	Compromised adhesion networks and impaired mechanotransduction	Cerrone et al., 2014
		*Dsg2* / p.D154E, p.D264E, p.N266S, p.D494N, p.A517V, p.G812S, p.G812C, p.C813R, and p.V920G	Reduced cell-cell cohesion	Force of detachment / shear stress	Desmoglein-2 interaction is crucial for cardiomyocyte cohesion	Schlipp et al., 2014
		*LMNA* / c.418_438dup (p. Leu140_Ala146dup)	Decreased mechanical resistance of the nuclear envelope	–	*LMNA* mutations cause decreased cardiomyocyte tolerance to mechanical stress	Forleo et al., 2015
	Murine ventricular myocytes	*Jup* 2057del2 over-expression	Increased apoptosis	Uniaxial cyclic stretch	Aberrant trafficking of intercalated disk proteins is a central mechanism in AC myocyte injury	Asimaki et al., 2014
	Neonatal rat ventricular myocytes	*DSP* / homozygous C-terminaltruncation	Altered morphology, elasticity, adhesion and viscoelastic properties	–	*DSP* mutations lead to cellular biomechanics impairment	Puzzi et al., 2018
	Human keratinocytes	*PKP2* / c.1760delT (p.V587Afs*655)	Accumulated lipid droplets, disarray of myofilaments	Physiological substrate stiffness and electrical stimulation	Cell-cell adhesion and mechanical sensing influences cell identity	Dorn et al., 2018
	hiPSC-CMs	*PKP2* / homozygous c.2484C > T	Lipid accumulation and apoptosis	Transplant into neonatal rat hearts	hiPSC maturation uncovers some of the AC phenotypes	Cho et al., 2017
		*PKP2* / homozygous c.2484C > T	Reduced gene expression response to mechanical stress	Physical confinement and cyclic uniaxial elongation	Transcriptional response to mechanical load is impaired	Martewicz et al., 2019
		*PKP2* / c.971_972InsT (p.A324fs335X/N)	Changes in gene expression, lipid accumulation, action potential shortening, re-entrant arrhythmia	Electrical stimulation	Syncytial constructs and matrix cues enable better modeling of heart tissue	Blazeski et al., 2019
		*PKP2* / homozygous c.2484C > T	Increased apoptosis and lipid accumulation	Hormonal treatment (testosterone)	Sex hormones can influence disease pathology	Akdis et al., 2017
		*DSG2* / p.G638R	Increased action potential shortening, arrhythmic events	Adrenergic stimulation	*DSG2* mutations influence cardiac ion currents and cause arrhythmia	El-Battrawy et al., 2018
		*TMEM3* / p.S358L	Decrease in the rising and decay time of Ca^2+^ transients, changes in contraction properties	Adrenergic stimulation	Isoprenaline induced abnormal Ca^2+^ transients	Padrón-Barthe et al., 2019
	hiPSC-cardiac microtissues	*PKP2* / c.2013delC (p.K672RfsX12)	Arrhythmias	Electrical stimulation	hiPSC-cardiac fibroblasts influence adjacent cardiomyocyte electrical behavior	Giacomelli et al., 2020

### *In vitro* Models of Mechanical Load in AC

Here we provide an overview of how mechanical load was integrated in cellular models to support the concept that stretch may impair cell adhesion, intracellular signaling, and intercellular conduction of excitation in AC cells.

#### Non-Human Models

New insights into the molecular basis of altered mechanosensing in AC have come from biophysical measurements in heterologous cell systems and murine cellular models. These systems proved useful in studying biomechanical properties of cells in the presence of altered or reduced desmosomal proteins.

The mouse atrial CM HL-1 cell-line has been widely used to study AC causing mutations. Overexpression of mutant PKP2 in HL-1 cells revealed that mutated PKP2 failed to interact with DSP (Kirchner et al., 2012), supporting the concept that mechanotransduction is impaired in AC (Mestroni and Sbaizero, 2018).

Atomic force microscopy (AFM) can be used to assess morphology and mechanical properties in single living cells, including cell-ECM interactions. Using a cantilever system, cell elasticity and viscoelasticity behavior toward externally applied forces can be measured, since alterations of cellular tension elements have an impact on cell stiffness. Pkp2 was knocked-down in these cells using short hairpin RNA (Puzzi et al., 2019) and single-cell force spectroscopy by AFM was used to determine cell-ECM interactions in Pkp2-deficient HL-1 cells. Specifically, Pkp2-knock-down was sufficient to induce changes in cytoskeleton organization with perturbation of the actin network and changes in focal adhesions. As a consequence, CM stiffness decreased, along with a reduction in work of detachment, suggesting an impact on CM-ECM interactions. Interestingly, the authors identified miR200b as one of the mediators of these effects, since miR200b predicted targets belong to the focal adhesion pathways. In line with this, downregulation of miR200b partially rescued the mechanical properties of Pkp2-deficient cells, with restoration of cellular stiffness but only partial actin network rescue, indicating that likely additional factors regulate the cytoskeleton organization.

HL-1 cells were also used to demonstrate that some Dsg2 mutations affect cell cohesion, which was measured using a liberase-based dissociation-assay. In addition, Dsc2 and N-cadherin interactions were measured using AFM and compared with cell-free single-molecule measurements (Schlipp et al., 2014).

Overexpression of mutated LMNA in HL-1 cells revealed decreased mechanical resistance of the nuclear envelope, again supporting the idea that one of the main pathological mechanisms in AC include decreased CM tolerance to mechanical stress (Forleo et al., 2015).

Among the murine cellular models, super-resolution fluorescence microscopy was used in single isolated mouse ventricular myocytes with *Pkp2*-heterozygous *null* genotype and identified increased separation between the microtubule plus end (marked by the protein EB-1) and N-cadherin. This supported the concept that defective desmosomal proteins interfere with adhesion networks resulting in impaired mechanotransduction properties of the cells (Cerrone et al., 2014).

Finally, a mutant *Jup* was overexpressed in neonatal rat ventricular myocytes that showed increased levels of apoptosis when subjected to brief intervals of uniaxial cyclical stretch for 4 h (Asimaki et al., 2014).

#### Human AC Cellular Models

Human tissue for studying AC is not easily obtained and it is limited to small biopsies, which are, however, mainly used for diagnostic purposes, and rare explanted hearts. From small biopsies, it is possible to isolate cardiac stromal cells that can be used to assess contribution to adipocyte differentiation (Sommariva et al., 2016; Pilato et al., 2018), while CMs isolated from biopsies cannot be kept very long in culture without de-differentiation. However, epithelial cells also express junctional and desmosomal proteins, therefore keratinocytes or buccal mucosa epithelial cells from AC patients represent an alternative cell source to investigate especially the localization of mutated proteins.

Similarly to the heart, the skin is a tissue that experiences continuous mechanical stress. It is therefore not unexpected that mutations in DSP, which is an essential component of cell-cell junctions both in CMs and keratinocytes, cause cardio-cutaneous syndrome (Carvajal syndrome; Norgett et al., 2000). Single cell force spectroscopy based-AFM was applied to human keratinocytes carrying a homozygous DSP mutation, which displayed altered morphology, elasticity, adhesion capabilities and viscoelastic properties compared to wild type keratinocytes, highlighting the tight interconnection between adhesion proteins and the intermediate filament scaffold (Puzzi et al., 2018).

After their discovery, human induced pluripotent stem cells (hiPSC) were soon differentiated into CMs and used to model several inherited cardiac diseases (Bellin et al., 2012; Giacomelli et al., 2017), including AC (Caspi et al., 2013; Kim et al., 2013; Ma et al., 2013). The majority of these models used hiPSCs from patients carrying mutations in *PKP2*, the most commonly mutated gene in AC. The first studies used hiPSC-CMs differentiated in embryoid bodies or in two-dimensional monolayer cultures, and the main focus of the AC phenotype was apoptosis and lipid accumulation. Importantly, under basic culture conditions, only some typical signs of the disease were detectable *in vitro*, including identification of defects of hiPSC-CMs in establishing cell-cell junctions (such as enlarged desmosomes at electron microscopy analysis, reduced immune-signal for desmosomal proteins). To uncover the AC phenotypes of CM apoptosis and lipid accumulation *in vitro* using AC hiPSC-CMs, both induction of metabolic maturation from glycolytic to fatty acid oxidation energetics was necessary and further co-activation of normal PPAR-alpha and abnormal PPAR-gamma pathways (Kim et al., 2013; Wen et al., 2015).

In standard CM cultures *in vitro*, the physical cues of the human heart are not sufficiently reproduced, and the capability of modeling the dynamic and highly organized environment of such a complex organ is limited. A well-recognized limitation of hiPSC-CMs is indeed their fetal-like phenotype, with disorganized sarcomeres and lack of T-tubules (where connexosomes are located) to mention some junctional proteins that are most relevant for AC modeling. Therefore, later studies used different strategies to reproduce the biophysical and mechanical characteristics typical of the heart, including mechanical load and stretch, continuous electrical stimulation, and anisotropic tissue organization.

By culturing 3-month old hiPSC-CMs under conditions mimicking the mechanical load of the heart (50 kPa substrate stiffness and 1 Hz electrical pacing), together with maturation/adipogenic medium, PKP2 mutant hiPSC-CMs not only accumulated lipid droplets, but also displayed disarrayed myofilaments, with progressive acquisition of fat cell identity (Dorn et al., 2018). This work highlighted the role of cell-cell adhesion and mechanical sensing in determining (cardiac) cell identity. This work is an exemplar of how changes in cell-cell contacts may induce changes in transcriptional programs and, in this particular case, control myocyte/adipocyte identity. Interestingly, by exome sequencing, the authors identified a mutation in the *MYH10* gene in a patient diagnosed with AC but without mutations in classical AC-causing genes. *MYH10* encodes the non-muscle myosin IIB (NMIIB) of the actomyosin cytoskeleton, which regulates both actin dynamics and accumulation of active RhoA-GTP at adherens junctions, thereby supporting the role of the mechanosignaling cascade in the pathogenesis of AC. hiPSC-CMs were also generated from this patient and they recapitulated AC traits *in vitro*, including reduced levels of NMIIB protein at the cell membrane, accompanied by decreased membrane RhoA and compromised cell-cell junctions. Importantly, the link between RhoA regulation by PKP2 and impact on CX43 was previously demonstrated in AC patients’ samples and HL-1 cells (Wang et al., 2015). However, a clear link between MYH10 and AC has not been firmly established yet. Notwithstanding, this work provided evidence that by using culture conditions more closely mimicking the mechanical strain of the heart, it was possible to reveal lineage conversion under pathological conditions and lipogenic stimuli.

Maturation to adult-like phenotype could be achieved by *in vivo* transplantation of *PKP2* mutated hiPSC-CMs into neonatal rat hearts, which allowed the recapitulation of lipid accumulation and apoptosis after 1 month (Cho et al., 2017). The elements that permitted maturation and development of AC pathological features in this experimental setting might be multifold and likely include microenvironment, cardiac-specific secreted factors, cardiac ECM, electrical pulses or even stiffness and mechanical load and stretch.

Cell-cell interactions play key roles in normal and pathological heart physiology and, electrophysiological and histopathological manifestations at tissue level may not be evident in single cells *in vitro*. To properly model these features *in vitro*, both precisely oriented syncytium-like structure and cyclic biomechanical stimuli must be accurately mimicked. Two multicellular syncytial models were used to study AC using hiPSC-CMs (Blazeski et al., 2019; Martewicz et al., 2019).

To specifically analyze how hiPSC-CMs reacted to mechanical load in the settings of AC, Martewicz et al. (2019) integrated both controlled substrate topology (50-μm wide linear confinement) and a stretching system to apply cyclic uniaxial elongation. Physical confinement resulted in a regular and organized sarcomeric structure, and in a more physiological distribution of desmosomal proteins, i.e., PKP2 was mainly localized at inter-cellular junctions. Without stretching system, the main difference in gene expression between AC and control hiPSC-CMs was found in ECM genes (including collagen and fibronectin encoding genes), cell-cell communication and cell adhesion. When 60 min cyclic anisotropic (parallel to the pattern) stretch was applied, the very early response of both control and AC hiPSC-CMs to mechanical stress was identified. Importantly, control but not AC hiPSC-CMs upregulated genes belonging to the response to mechanical stimulus, such as Jun proto-oncogene, AP-1 transcription factor subunit (JUN) and FOS proto-oncogene, AP-1 transcription factor subunit (FOS). The design of this study allowed for the identification of early response genes, however, it would be informative to capture the adaptive changes by analyzing responses to different magnitudes and duration of stretch, and how biophysical parameters, such as cellular and tissue viscoelasticity properties impact cellular consequences of mechanical stimuli.

Engineered heart slices built by seeding AC hiPSC-CMs (carrying a *PKP2* mutation) in de-cellularized porcine myocardium sections (Blazeski et al., 2019) which within 2 weeks formed spontaneously beating multi-layered syncytia, with aligned and sometimes multinucleated CMs, organized sarcomeric structures and elongated nuclei. The engineered slices could be stimulated at different frequencies (namely from 0.5 to 2 Hz). In these settings several AC-related features were confirmed. In particular, anisotropic propagation of contraction allowed for the identification of re-entrant arrhythmias. This is a disease-relevant phenotype of important physiological significance that could not be recapitulated in other standard culture formats.

### *In vitro* AC Models of Adrenergic Stress

Several *in vitro* cell models were used to address the electrical aspects of AC and some tried to recapitulate the effects of adrenergic stimulation.

Among these, *DSG2*-mutated hiPSCs-CMs were used to investigate the connection between desmosome mutations and arrhythmia (El-Battrawy et al., 2018). Electrophysiology was the main focus in this study and AC hiPSC-CMs displayed reduced I_Na_ impacting on a slower upstroke velocity of the AP. Furthermore, I_Na_, I_NCX_, I_to_, I_SK_, and I_KATP_ were decreased, while I_Kr_ was enhanced in AC hiPSC-CMs compared with control hiPSC-CMs. Interestingly, AC hiPSC-CMs were more sensitive to adrenergic stimulation by isoprenaline, which caused a more pronounced AP shortening but also a higher incidence of arrhythmic events (both early-after depolarization and delayed after depolarizations). These results support the hypothesis that enhanced AP shortening by adrenergic stimulation may increase arrhythmia susceptibility in this cell model, thus recapitulating what happens in AC patients under catecholamine stress, where enhanced AP shortening increases the propensity to arrhythmias.

Human induced pluripotent stem cells with mutated TMEM43 were generated by CRISPR/Cas9 (Padrón-Barthe et al., 2019). Although TMEM43 is not a protein of the desmosome but a transmembrane protein of the nuclear envelope, hiPSC-CM stimulation with isoprenaline caused a decrease in the rising and decay time of the Ca^2+^ transients. Interestingly, contraction properties of the mutated iPSC-CMs were changed under basal conditions. GSK3β inhibition using CHIR99021 partially rescued the contraction properties. It is interesting to note that GSK3β is both a crucial component of the Wnt/β-catenin pathway, which has been shown to play a crucial role in AC (Lorenzon et al., 2017), and a central regulator of the β-adrenergic response (Zhou et al., 2010). Interestingly, hiPSCs with truncation mutations in PKP2 showed reduced immunofluorescence signal for PKP2 itself, CX43, Nav1.5 channel, and SAP97 (Asimaki et al., 2014), features that were reverted by treatment with another GSK3β inhibitor (SB216763).

As discussed above, early AC hiPSC-CM studies used media compositions able to activate both PPAR-alpha and PPAR-gamma pathways (Caspi et al., 2013; Kim et al., 2013; Ma et al., 2013). Furthermore, despite adrenergic stimulation not being directly addressed in these studies, it is worthwhile noting that some compounds in the lipogenic media not only impact cell metabolism, but might also have modulated cAMP levels (dexamethasone, IBMX, which in turn is a second messenger belonging to the signaling cascade of adrenergic stimulation), and β-adrenergic receptors (insulin and rosiglitazone). This highlights a major limitation of studies in hiPSC-CMs using adipogenic induction protocols which are inherently artificial in composition and have different time-scales. Indeed, fibro-fatty substitution in the AC myocardium progresses over years to decades and is likely incrementally influenced by various prevailing endogenous and exogenous factors.

Arrhythmogenic Cardiomyopathy shows gender imbalance and it has already been shown for other cardiovascular diseases that there might be a direct role of sex hormones in influencing disease pathology and arrhythmogenesis. In line with this, AC hiPSCs generated by Kim and colleagues (carrying a *PKP2* homozygous mutations) were used to show that testosterone was increased while estradiol decreased apoptosis and lipid accumulation in AC hiPSC-CMs (Akdis et al., 2017). This is interesting as some studies suggested that testosterone has pro-arrhythmic effects by modulating cardiac contraction and Ca^2+^ homeostasis whereas estradiol has anti-arrhythmic effects. In addition, arrhythmias may occur due to adrenergic-induced apoptosis which would worsen AC pathophysiology (Veldhuis et al., 2009; Tsai et al., 2014).

There is a close functional association between NaV1.5 and mechanical junctional proteins, supported by NaV1.5 co-precipitation with PKP2 (Cerrone et al., 2012) and with N-cadherin (Sato et al., 2011). This supports the presence of an adhesion/excitability node in cardiac myocytes. hiPSC-CMs carrying the p.R1898H missense substitution variant showed reduced I_Na_ density compared with isogenic corrected controls and reduced density of NaV1.5 and N-cadherin clusters at the site of cell contact (Te Riele et al., 2017). NaV1.5 might then not only be a cardiac ion channel, but also a multifunctional protein in an active adhesion/excitability complex with mechanical junctions that orchestrates the interaction between mechanical and electrical junctions (Te Riele et al., 2017). Lastly, the link between mutated PKP2 and I_Na_ has also been shown (Cerrone et al., 2014).

We have recently described a novel cardiac microtissue model entirely derived from hiPSCs, where fixed ratios of hiPSC-CMs, -cardiac fibroblasts and -cardiac endothelial cells were combined in a 3D spheroid structure. To mimic the high frequency ranges that are reached following beta-adrenergic stimulation during physical exercise, these microtissues were paced at increasing beat frequencies. Interestingly, replacing wild-type with PKP2-defective hiPSC-cardiac fibroblasts was sufficient to induce an arrhythmic phenotype in the cardiac microtissues, despite the CMs being healthy (Giacomelli et al., 2020). This illustrated the crucial role that cardiac fibroblasts, although non-excitable themselves, have in modulating active and passive electrical properties of adjacent CMs (Gaudesius et al., 2003; Miragoli et al., 2006; Ongstad and Kohl, 2016) and that PKP2-defective cardiac fibroblasts were integral contributors to the AC phenotype. The mechanism for the arrhythmic behavior could be related to the overall reduced CX43 expression and/or remodeling observed throughout cardiac microtissues containing AC hiPSC-cardiac fibroblasts, even though further studies are needed to clearly identify the histological and functional effects of these heterocellular interactions *in vitro*.

### Limitations of AC *in vitro* Cellular Models and Possible Solutions

Although *in vitro* cellular models have contributed to our understanding of the pathogenic mechanisms underlying AC, including genetic factors, cellular contributions, signaling pathways, and molecular defects, they also bring some limitations that need attention.

First, isolated cells lack the physiological realism of *in vivo* tissues, where different cell types are organized in functional units and both homo- and hetero-cellular interactions contribute to tissue homeostasis, including biophysical, mechanical, and electrical properties. Importantly, cell-cell communication is essential for the proper propagation of electrical impulses and mechanical strains. Second, cell-ECM interactions are essential for maintaining tissue structure and dynamics, where the ECM contributes to heart stiffness, which includes viscosity and elasticity. Furthermore, ECM is a crucial organizer of the cellular microenvironment, where hormones, growth factors, and other molecules travel in the extracellular space (Valiente-Alandi et al., 2016). This brings us to the third limitation of *in vitro* cellular models, the lack of systemic regulatory stimuli such as cytokines, hormones, neuro-hormones, and microRNAs that can act in both autocrine and paracrine manners. Finally, some specific clinical features, like predisposition of the right or left ventricle as seen in certain AC patients and the appearance of re-entrant arrhythmia are difficult to model and recapitulate *in vitro*.

Multicellular interactions play key roles in heart physiology and pathophysiology, but *in vitro* models often include only one cell type. Some pathological signs might depend or develop only in the presence of cell-cell interactions, and their electrophysiological and histopathological manifestations at the tissue level may not be evident in single cells. It is then still difficult that all the aspects of the disease can be faithfully studied *in vitro* in one cell type in isolation, but solutions are starting to emerge.

A key step in the field is the development of micro-environments that more closely mimic the bio-physical properties of the native heart, by employing biomechanical devices able to integrate physical cues in biological models, providing tools to better understand *in vivo* environment.

Some limitations are restricted to hiPSC tools and are discussed here. In the majority of the studies, hiPSCs from unrelated healthy donors were used as controls, however, it is known that a high degree of variability is observed in iPSC-CMs from different hiPSC lines (Volpato and Webber, 2020), and even among wild-type control hiPSC-CMs, especially with regards to AP properties (Sala et al., 2017) but also cardiac ion currents (Hoekstra et al., 2012). The solution here is to use gene-corrected hiPSCs (Meraviglia et al., 2020) as a control or introduce the mutation of interest in a wild-type hiPSC line (Padrón-Barthe et al., 2019). Another limitation well-known to the hiPSC community is the immaturity of hiPSC-CMs. Ion channels are indeed not expressed at the same levels as adult cardiac myocytes, sarcomeres are disorganized, contraction force is small with non-physiological force-frequency relationship, mitochondria-to-cell volume is low, and energetics mainly rely on glycolysis versus beta-oxidation of fatty acids (Hoekstra et al., 2012; Veerman et al., 2015). Therefore hiPSC-CMs may not be completely representative of diseases (such as AC) with adolescence-adulthood onset. In this respect, methods to enhance structural, mechanical, electrical and metabolic maturation of hiPSC-CMs are all useful for obtaining meaningful insights.

Exercise-like conditions are difficult to reproduce comprehensively *in vitro*, but electrical stimulation, mechanical stretch, or substrate stiffness change and addition of pharmacological compounds can be used to investigate the effects of individual aspects of exercise physiology, such as cardiac muscle contraction or activation of exercise-responsive signaling pathways, similar to what is performed for skeletal muscle (Carter and Solomon, 2019). Whilst a few studies using AC hiPSC-CMs have used such an approach, to our knowledge other AC cellular systems have not been challenged in this way. In the future, it will be important to identify the exact metabolic, inflammatory, and signaling changes induced by exercise *in vivo*, to be able to mimic those *in vitro*. Indeed, “exercise-in-a-dish” approaches allow investigations into different aspects of exercise with a level of abstraction not possible *in vivo*, either directly upon a specific cell type, or as exercise-mediated cross-talk between different cell types.

Cardiac tissue engineering may provide a solution to the challenge of faithfully recapitulating *in vitro* factors that are recognized as playing a role in AC pathogenesis, as discussed above. Ideally, advanced systems will be needed, where distinct cardiac cell types are organized in multicellular three-dimensional syncytia, built using cardiac-specific ECM, and subjected to physiological mechanical load (cyclic stretch and contraction against resistance) and electrical stimulation. In this respect, recent advancements in hiPSC technology provide precious resources for investigating electromechanical training in AC such as: cardiac tissues that can be subjected to physical conditioning with increasing intensity over time (Ronaldson-Bouchard et al., 2018); tissue slices similar to those generated by Blazeski et al. (2019) but replacing porcine with human ECM and higher frequency stimulating to mimic strenuous exercise; engineered heart tissues of different formats which enhanced I_Na_ density (Lemoine et al., 2017; Tiburcy et al., 2017), *t*-tubule formation together physiological contractile force responses (Mannhardt et al., 2016), and inotropic responses to β-adrenergic stimulation mediated via canonical β1- and β2-adrenoceptor signaling pathways (Tiburcy et al., 2017); thin cardiac muscle films (together with optogenetics and optical mapping) and high pacing rate and β-adrenergic stimulation to uncover re-entry arrhythmias as rotors in these tissues (Park et al., 2019).

## AC Animal Models

In cardiovascular research, *in vivo* models mainly include rodent models (Camacho et al., 2016) and the teleost zebrafish which have become increasingly important for studying developmental cardiovascular diseases (Beffagna, 2019).

The discovery of genes linked to AC and advances in genetic engineering made it possible to create transgenic, knock-in, and cardiac-specific knockout animal models for both desmosomal and non-desmosomal proteins (for a comprehensive overview, readers are referred to Gerull and Brodehl, 2020). These models contributed to confirm AC causative roles for many gene mutations and provided novel mechanistic insights into AC pathogenesis. The advantage of animal models is that they provide a defined genetic background which allows to clearly identify the cause of AC. However, some limitations apply to animal models, especially with regards to disease onset, progression, and prognosis that in patients are influenced by a variety of factors including environment, comorbidities, age, genetic background, and epigenetic factors, which are difficult to recapitulate in animals. As an example, mice and zebrafish do not show fatty substitution of the myocardium, one of the hallmarks linked to AC progression in humans.

Importantly, animal models represent a precious tool to help unravel the molecular mechanisms underlying AC in relation to mechanical load and adrenergic stimulation. Under physiological conditions, the heart is constantly challenged by cyclic mechanical stress. The study of many AC models at rest, i.e., without exercise simulation etc., suggested that AC phenotype, although genetically determined, develops postnatally and progresses with age, probably due to the temporal action of cardiac tissue extrinsic and/or intrinsic mechanical forces. While exercise training in these animals highlighted the role of physical stress in accelerating disease onset and precipitating arrhythmic events.

Here we focus on *in vivo* models that investigated the connection between AC and physical exercise, mechanical load and adrenergic stimulation (Table 3). More general overviews on all AC animal models can be found in (McCauley and Wehrens, 2009; Pilichou et al., 2011; Padrón-Barthe et al., 2017; Austin et al., 2019).

### Physical Exercise and Mechanical Load in AC Animal Models

Research on the effects of exercise and adrenergic signaling as a trigger for AC pathological phenotype is limited by the experimental difficulties in implementing training protocols for animal models. Nevertheless a few studies successfully linked strenuous physical activity and accelerated phenotype progression in AC.

Homozygous mice with a germline Pkp2 deficiency showed severe heart defects during cardiac development due to reduced architectural stability of the intercalated disks (Grossmann et al., 2004). However, the heterozygous mouse was viable and three- or six-month-old Pkp2 heterozygous mice displayed no structural or electrical abnormalities but arrhythmic susceptibility to flecainide. In Pkp2 heterozygous mice, protein levels of Ca^2+^ handling proteins were reduced compared to wild-type siblings. However, when 12-week-old mice were subjected to 1-month voluntary running on a treadmill, their hearts showed lateralization of Cx43 in right ventricular myocytes, right ventricular conduction slowing, and a higher susceptibility toward arrhythmias (van Opbergen et al., 2019). This suggested a cross-talk between the desmosomal integrity and Nav1.5 complexes and a possible contribution of sodium current (I_Na_) dysfunction to arrhythmias. Moreover, exercise induced a pro-arrhythmic cardiac remodeling based on impaired Ca^2+^ cycling and electrical conduction. As for structural remodeling, physical exercise exacerbated the fibrotic response.

A novel PKP2 R735X nonsense mutation dominant-negative mouse model was generated using adeno-associated virus gene delivery. Expression of the mutant protein was induced after a single AAV9-R735X intravenous injection and stable cardiac expression of mutant Pkp2 was achieved 4 weeks after. Endurance exercise training (swimming endurance training protocol) was started 2 weeks later. After 10 months of strenuous swimming, trained but not sedentary one-year-old mice developed RV regional and global dysfunction, as well as an altered localization and punctate distribution of Cx43 at intercalated disks (Cruz et al., 2015).

Different mouse models were generated to mimic Naxos disease, showing defects in embryonic skin architecture and extreme sensitivity to mechanical stress (Bierkamp et al., 1996, 1999; Zhang et al., 2015). Bierkamp and colleagues demonstrated that plakoglobin *null* mutant embryos with a 129/Sv genetic background, died due to severe heart defects, starting from embryonic day (E) 10.5. Histological sections of E10.5 and E12.5 embryos revealed that mutant hearts were structurally less well developed, with thin and weak walls. Coagulated blood was often found in atria, ventricles, and pericardium, suggesting cardiac dysfunction. In a different genetic background, such as C57BL/6, embryos developed and died around birth, due to cardiac dysfunction and to a severe skin phenotype. The superficial layer of the epidermis in different body districts was detached, leading to regions with a very thin epidermis. The skin showed altered physical properties; it dried more quickly and was extremely sensitive to mechanical stress such as rubbing and cutting (Bierkamp et al., 1996). Unfortunately, mutant mice die during late embryogenesis or soon after birth, indicating that there could be differences in the mutant *JUP* expression levels between human patients and mouse models. Interestingly, a stable zebrafish model of AC with CM-specific expression of the human *JUP* 2057del2 mutation responsible for Naxos disease was created in order to study the pathogenesis of the disease (Asimaki et al., 2014). By 4 to 6 weeks of age, the mutant animals showed heart enlargement with marked thinning of atrial and ventricular walls, cachexia, peripheral edema, and high mortality. A reduction of the I_Na_ current density was also described. This stable AC zebrafish model was used in a chemical screen leading to the identification of a small molecule named SB216763 that was able to rescue AC phenotypes (Asimaki et al., 2014). SB216763 is described as an inhibitor of GSK3β, increasing canonical Wnt/β catenin signaling (Coghlan et al., 2000). Interestingly, SB216763 was subsequently used to prevent myocyte injury and cardiac dysfunction in two AC mouse models both at baseline and in response to physical exercise (Chelko et al., 2016). In this study the following mice were used: (i) a mouse model related to the zebrafish model described above with transgenic overexpression of mutant *Jup* 2057del2 mutation, encoding human Naxos JUP, named Jup2157del2 and (ii) another mouse model, named Dsg2 mut, the loss of exons 4 and 5 of murine *Dsg2*, causes a frameshift mutation and premature termination of translation. Mice began SB216763 treatment at 3 weeks of age, and a subset of these mice began a gradually incremented exercise training protocol (swimming) at 5 weeks of age. Heterozygous Dsg2mut/+ mice did not show an overt AC phenotype at rest but developed myocyte injury and redistribution of ID proteins in response to endurance exercise. Importantly, activation of a common disease pathway in Dsg2mut/+ animals subjected to swimming was blocked by SB216763. Treatment with SB216763 improved cardiac function, myocardial injury, and survival in exercised mice, implicating a central role for GSK3β signaling in the pathogenesis and progression of AC in response to exercise (Chelko et al., 2016).

While systemic *Dsp-null* mutations but also cardiac-specific overexpression of heterozygous V30M or Q90R mutant *Dsp* resulted in embryonic lethality (Gallicano et al., 1998; Yang et al., 2006), cardiac-specific overexpression of the C-terminal mutant R2834H *Dsp* resulted in viable mice that developed ventricular enlargement and biventricular cardiomyopathy. This last transgenic cardiac-specific model was used by Martherus and colleagues to investigate how chronic endurance exercise may lead to AC pathogenesis. Transgenic mice that overexpressed wild-type or R2834H mutant DSP (Tg-Dsp^WT^ or Tg-Dsp^R2834H^) along with control non-transgenic (NTg) littermates were kept sedentary or exposed to a daily running regimen for 12 weeks. Accelerated cardiac remodeling was evident upon exercise in comparison with non-exercised mice and mutant animals showed RV dilation and focal fat infiltration whereas cardiac function was preserved in NTg and Tg-Dsp^WT^ littermates. Hearts obtained from trained Tg-Dsp^R2834H^ mice showed focal fat infiltrations in the right ventricle, cytoplasmic aggregations consisting of Dsp, Jup, and Cx43 and the disruption of the intercalated disks, intermediate filaments, and microtubules (Martherus et al., 2016). These results confirmed the role of physical training in precipitating cardiac remodeling and dysfunction in AC.

The AC mouse model carrying a cardiac-specific disrupted form of DSP did not show any discernible AC phenotype within the first couple of months after birth but exhibited cardiac systolic dysfunction and myocardial fibrosis starting from 6 months of age which correlated with plakoglobin delocalization to the nucleus where it is able to suppresses Wnt/β-catenin canonical signaling (Garcia-Gras et al., 2006). Intriguingly, treadmill exercise in these mice did not accelerate AC progression, rather it restored transcript levels of the majority of dysregulated genes in CMs (particularly those involved in inflammation, epithelial to mesenchymal transition (EMT), and oxidative phosphorylation), reduced apoptosis, and induced cardiac hypertrophy without affecting cardiac function (Cheedipudi et al., 2020). It is important to note that these findings should not be interpreted to encourage sport activity in AC patients, rather they raise the hypothesis that only strenuous exercise and not physical activity *per se* is detrimental in AC. Indeed, these *Dsp* mutant mice were exposed to 60-min regular treadmill run for 3 months. In the future it will be important to include short- and long-term treadmill exercise in AC models to discern between the molecular mechanisms involved in onset and acceleration of the disease phenotype from protective mechanisms linked to mechanical load.

Lyon and colleagues generated a CM-specific *Dsp*-knockout mouse model using a ventricular myosin light chain-2-Cre construct. Homozygous *Dsp*-knockout mice were viable but showed early ultrastructural defects in desmosomal integrity leading to a biventricular form of AC. The myocardium alterations included cell death and fibro-fatty replacement within the ventricle leading to biventricular dysfunction, heart failure and premature death. In these mice, to establish if in *Dsp*-cKO hearts the loss of Cx43 is a primary or secondary consequence of Dsp depletion, authors evaluated the dose-dependent effects of loss of DSP versus controls, analyzing Cx43, plakophilin-2 and N-cadherin in neonatal CM cultures generated from Dsp-floxed mice. They demonstrated that the level of Cx43: (i) follows the dose-dependent knock-down of DSP and (ii) is found independent from any molecular dissociation of the desmosomal and fascia adherens junction complex, as evidenced by the maintained levels of plakophilin-2 and N-cadherin in neonatal CMs following DSP knock-down (Lyon et al., 2014). The down regulation of Cx43 in this mouse model, results in conduction abnormalities prior to mechanical junction complex damage, and fibro-fatty replacement (Lyon et al., 2014). Interestingly, these mice exhibited ventricular arrhythmias that were exacerbated with exercise and catecholamine stimulation, supporting the idea that vulnerability to adrenergic stress can be captured with AC animal models.

In order to study AC, different zebrafish models have also been used (Heuser et al., 2006; Martin et al., 2009; Moriarty et al., 2012) without a specific training protocols. However, unlike mouse models, fish swim actively throughout their life even if they are not forced to swim-training. *Dsp*-knock-down zebrafish models were generated with antisense morpholino, confirming specific and disruptive effects on desmosomes, like those identified in AC patients (Giuliodori et al., 2018). Dsp-deficient zebrafish models were used for an *in vivo* cell-signaling screen, using pathway-specific reporter zebrafish lines. This work demonstrated that Wnt/β-catenin, TGFβ/Smad3, and Hippo/YAP-TAZ pathways were significantly altered in AC zebrafish models, with Wnt as the most dramatically affected. Involvement of Wnt/β-catenin signaling in the pathogenesis of AC (Garcia-Gras et al., 2006) and the ability of mechanotransduction to activate canonical Wnt/β-catenin signaling (Warboys, 2018) was also confirmed in zebrafish. Furthermore, other authors demonstrated that YAP/TAZ play a central role in delivering information of mechanical environments surrounding cells to the nucleus transcriptional machinery suggesting a link between mechanotransduction and hippo pathway (Dobrokhotov et al., 2018). Interestingly, under persistent Dsp deficiency, Wnt signaling is rescuable both by genetic and pharmacological approaches, which may suggest new therapeutic scenarios (Giuliodori et al., 2018).

The study of AC molecular mechanisms has also made use of spontaneous animal disease models. AC is a spontaneous and manifest disease in the Boxer breed of dogs with striking histopathological, anatomical, genetic, and biomolecular similarities with AC in humans (Vischer et al., 2017).

Proprietary Boxer dogs are free to move and thus subjected to exercise-induced stimuli. Exposure to risk factors similar to those of humans may be one reason for spontaneous AC occurrence. The evidences for Striatin gene (*STRN*) as causative gene for AC canine disease is still under debate (Meurs et al., 2010; Cattanach et al., 2015). Intriguingly, STRN co-localizes with desmosomal proteins, interferes with the Wnt pathway and is involved in intracellular Ca^2+^ regulation (Oxford et al., 2014; Montnach et al., 2018), thus potentially contributing to mechanotransduction and downstream adrenergic signaling.

In 2000 a spontaneously occurring cardiac disease in domestic cats was reported, that shared remarkable similarities with human AC, both for clinical and pathological features (Fox et al., 2000). The histopathological analysis of the cats’ hearts showed evidence of myocardial cell injury and cell-death as well as repair in the right ventricle, closely resembling AC patients. Subsequent reports described two AC cats with right predominant AC (Harvey et al., 2005), and an AC cat model with severe left ventricular involvement (Ciaramella et al., 2009). This feline model is considered a potentially important investigative tool that could enhance the understanding of the complex clinical and pathophysiological AC mechanisms, as well as the genetic factors and molecular mechanisms responsible for its genesis.

PLN is a crucial regulatory protein for Ca^2+^-cycling and an important mediator of the adrenergic effects resulting in enhanced cardiac output. A mouse model with cardiac-specific expression of the heterozygous human PLN Arg14Del mutation recapitulated the human phenotype and suggested the inhibition of SERCA2a activity, likely mediated through a disturbance in the structure of PLN, as a mechanism of action (Haghighi et al., 2006). Interestingly, phospholamban content in murine was increased in denervated skeletal muscles (Komatsu et al., 2018). Cardiac sympathetic denervation represents a well-established intervention in patients with ventricular arrhythmias refractory to pharmacotherapy and ablation, especially long QT-syndrome, but its role in other cardiomyopathies including AC is less clear (Coleman et al., 2012; Schwartz, 2014). Nevertheless, when applied to mouse models, this technique could help understanding the role of innervation in the progression of AC. It is interesting to note that the interface between neuron and CMs, also called neuro-cardiac junction, could explain the heart ability to function with precision, specificity and elevated temporal resolution to mechanical stretch and adrenergic stimulation in mouse models (Zaglia and Mongillo, 2017). Interestingly, *ex vivo* murine hearts were used to demonstrate the existence of a tight link between impaired desmosomal binding and a reduced response to adrenergic stimulation by isoprenaline, probably *via* disruption of β1-adrenergic receptor localization (Schlipp et al., 2014).

Altogether, these studies support the concept that in the presence of AC causing mutations, exercise is a trigger associated with aggravation of AC phenotype and a more rapid progression of the disease. However, it is worthwhile mentioning that, in line with some human studies, certain pre-clinical models also support the idea that extreme exercise may cause AC-like changes, even in the absence of a genetic predisposition. In male Wistar rats without known AC gene mutations, vigorous running exercise alone was able to induce fibrosis in the right ventricle and predispose to right ventricular arrhythmias with programmed electrical stimulation (Benito et al., 2011).

Finally, evidence that inflammatory mediators might play an important role in AC onset and progression are leading to exploring anti-inflammatory drug therapy as a potential effective strategy to reduce myocardial damage and risk of SCD. In Dsg2-mutant mice, anti-inflammatory treatment through inhibition of the NFκB signaling pathways was effective in reducing the structural and functional signs associated with AC disease progression (myocardial fibrosis, necrosis, inflammation, and arrhythmias; Chelko et al., 2019). Moreover, cytokines implicated in granulomatous inflammation led to intracellular translocation of junctional plakoglobin in cultured neonatal rat ventricular myocytes (Asimaki et al., 2011). Emerging evidence linking systemic inflammatory stress with perturbed desmosome function in both heart and skin (Paller et al., 2018) and the increasing understanding of the interplay between inflammation and physical exercise, might provide further insights into the mechanisms involved in AC disease progression due to exercise (Elliott et al., 2019).

## Conclusion

Exercise is one of the main triggers for life-threatening arrhythmias and SCD in several conditions that are vulnerable to adrenergic stress, in particular inherited diseases syndromes including AC (Cerrone, 2018). In the setting of AC, this can be mechanistically linked to: (i) a direct mechanical stress in cells with weakened cell-cell junctions, (ii) adrenergic surge, and (iii) electrophysiological remodeling.

**FIGURE 3:**
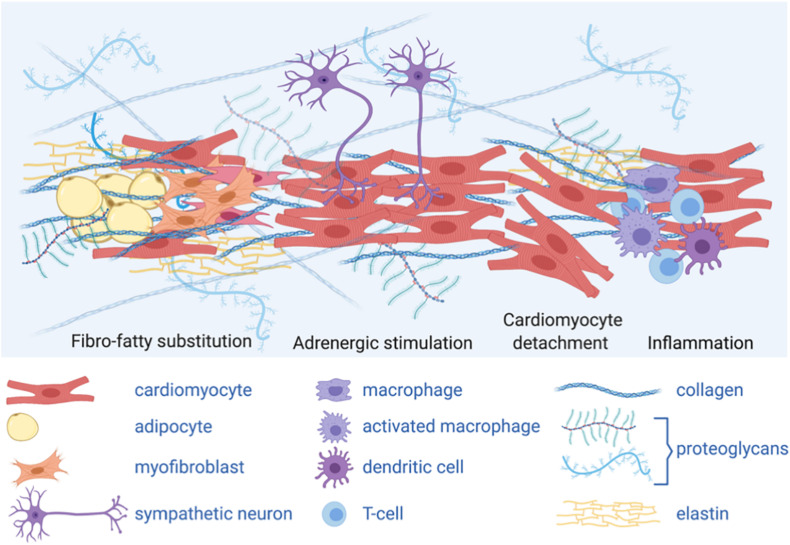
A complex extracellular environment influences intracellular signaling in AC. Graphical representation of different cell types and extracellular matrix (ECM) composing AC heart micro-environment: adipocytes and ECM protein deposition, including collagen, secreted by activated fibroblasts (myofibroblasts) change the stiffness of the heart tissue, thus impacting mechanical stimuli; fibro-fatty substitution is likely secondary to cardiomyocyte damage and death; sympathetic neurons release catecholamines, acting as adrenergic stimuli; inflammatory cells are also present in the AC myocardium. The complex cardiac micro-environment is sensed by the cells and influences intracellular signaling and cell fate.

Importantly, a complex extracellular environment influences intracellular signaling in AC (Figure 3). Structural weakening of the desmosome is a hallmark of AC pathogenesis, which predisposes the right ventricle to fibrosis and dilation (Stokes, 2007). The underlying molecular mechanisms include impairment of the mechanical CM-CM and CM/non-CM junctions, released desmosomal transcription regulators and adipogenic differentiation of mesenchymal cells. Both inhibition of canonical Wnt/β-catenin and activation of the Hippo signaling pathways have emerged as active players in AC pathogenesis. Interestingly, the Hippo pathway, which responds to cell polarity and mechanotransduction, is able to regulate cell proliferation, apoptosis and cell fate.

Activation of the adrenergic signaling cascade is a precipitating event which is involved in different forms of heart failure, as β-receptor blockers are a favorable pharmacological treatment of heart failure (Waagstein, 1993; Port and Bristow, 2001; Ponikowski et al., 2016). Furthermore, adrenergic signaling is a worsening factor for primary arrhythmic syndromes, such as long-QT syndrome, where left cardiac sympathetic denervation represents a therapeutic option (Schwartz et al., 2004).

In the early concealed stages, electrical abnormalities can be observed in the absence of overt structural changes, such as fibro-fatty replacement of the myocardium (Kaplan et al., 2004b; Rizzo et al., 2012).

Importantly, the I_Na_ current depends on the expression and structural integrity of desmosomes. A clear link between proteins of the desmosome and electrical stability of CMs is evident by several evidence that show reduced I_Na_ in the presence of reduced or mutated PKP2 and DSP and explain how impaired mechanical coupling may affect electrical properties of CMs (Zhang et al., 2013). In this view, correction of the mechanical coupling or removing the external stressor of mechanical load could prevent electrical dysfunction.

Heart failure in general has been associated with both elevated sympathetic tone and mechanical load (Lohse et al., 2003). Both systems activate signaling transduction pathways that increase cardiac output, but adversely contribute to electrical stability, at least partially *via* modulation of Ca^2+^ handling. PKA and CaMKII are key to the regulation of L-type Ca^2+^ channels and RYR2 in the β-adrenergic and stretch response. PKA targets L-type Ca^2+^ and RYR2 *via* A-kinase anchoring proteins, and transmits signals from β-adrenergic receptors *via* cAMP (Catterall, 2015; Landstrom et al., 2017).

In the early concealed stages of the disease, AC patients can be asymptomatic even if they are at high risk for SCD. The study of such patients is hampered by the difficulty in obtaining cardiac samples at the early stages of AC disease. Both *in vitro* cellular and *in vivo* animal models represent precious tools for modeling also the early stages of the disease to identify the initial molecular events triggering disease manifestation and therefore identify novel potential targets for therapies. Indeed, while AC manifestation progresses over the years in patients, documented early changes in AC animal models include electrical remodeling and electrical instability, along with desmosome loosening. Advanced technology including sophisticated intravital imaging systems applied to animal models and tissue engineering to mimic the highly organized 3D multicellular structure of the heart have the potential to advance our knowledge of AC pathogenesis, including untangling the link between physical exercise/adrenergic stress and molecular/electrical changes. Although complementary studies in AC human patients will be required, the need for novel therapeutic options that can prevent disease manifestation remains an open challenge and is one of the most important tasks to address to improve patients’ survival.

## Author Contributions

GB, ES, and MB conceptualization, writing original draft, and reviewing. All authors contributed to the article and approved the submitted version.

## Conflict of Interest

The authors declare that the research was conducted in the absence of any commercial or financial relationships that could be construed as a potential conflict of interest. The handling editor declared a past co-authorship with one of the authors GB.

## Abbreviations

AA: Aortic Aneurism
AC: Arrhythmogenic Cardiomyopathy
ACC: American College of Cardiology
ACTN1: α-actinin -1
ACTN2: α-actinin -2
AFM: Atomic force microscopy
AHA: American Heart Association
AP: Action potential
BS: Brugada Syndrome
Ca^2+^: Calcium
CaMKII: Calcium/calmodulin-dependent protein kinase II
cAMP: Cyclic AMP
CDH2: N-caderin (Cadherin-2)
CM: Cardiomyocyte
CPVT: Catecholaminergic Ventricular Tachycardia
CRISPR/Cas9: Clustered Regularly Interspaced Short Palindromic Repeats/CRISPR associated protein 9
CTNNA3: α T-catenin
CX43: Connexin 43
DC: Dilated Cardiomyopathy
DES: Desmin
DSC: Desmocollin
DSG: Desmoglein
DSP: Desmoplakin
E: Embryonic day
ECM: Extracellular matrix
EDMD: Emery-Dreifuss muscular dystrophy
EMT: Epithelial-mesenchymal transition
FLNC: Filamin C
FOS: Fos proto-oncogene, AP-1 transcription factor subunit
GSK3b: Glycogen synthase kinase 3 beta
HC: Hypertrophic Cardiomyopathy
Hippo: Salvador-Warts-Hippo signaling pathway
hiPSC: Human induced pluripotent stem cells
HRS: heart rhythm society
I_KATP_: K -selective, inward rectifier current
I_Kr_: Rapidly Activating Delayed Rectifier Potassium current
I_Na_: Voltage-gated sodium current
I_NCX_: Sodium/Calcium exchanger current
I_SK_: Small conductance Calcium-activated potassium current
I_to_: Transient outward potassium current
JUN: Jun Proto-Oncogene, AP-1 Transcription Factor Subunit
JUP: Plakoglobin
LDB3: LIM Domain Binding 3 (ZASP)
LDS: Loeys-Dietz syndrome
LMNA: Lamin A/C
LVNC: Left Ventricular non-compaction
miR: microRNA
MYH10: Myosin Heavy Chain 10
Nav1.5: Cardiac sodium channel
NMIIB: Non-muscle myosin IIB
NTg: Non-transgenic
PKA: Protein kinase A
PKP: Plakophilin
PLN: Phospholamban
PPAR: Peroxisome proliferator-activated receptor
RCM: Restrictive Cardiomyopathy
ROCK: Rho-associated protein kinase
RYR2: Ryanodine Receptor-2
SAP97: Synapse-associated protein 97
SCD: Sudden cardiac death
SCN5A: Sodium voltage-gated channel alpha subunit 5
SERCA: Sarco-Endoplasmic Reticulum Calcium ATPase
Smad: Small mother against decapentaplegic
STRN: Striatin
TAZ: Tafazzin
Tg: Transgenic
TGF β 3: Transforming growth factor β 3
TJP1: Tight Junction Protein ZO-1
TMEM43: Transmembrane protein-43
TTN: Titin
Wnt: Wingless-type pathway
YAP: Yes-associated protein.
